# Investigation of the Performance of Hastelloy X as Potential Bipolar Plate Materials in Proton Exchange Membrane Fuel Cells

**DOI:** 10.3390/molecules29061299

**Published:** 2024-03-14

**Authors:** Jiacheng Zhong, Zimeng Liu, Meng Zhang, Feng Liu, Wenjin Li, Beirui Hou, Wenmin Zhang, Chunwang Zhao, Mingxing Gong

**Affiliations:** 1 School of Materials Science and Hydrogen Energy, Foshan University, Foshan 528000, China; zjc160403201@163.com (J.Z.); 13432693515@163.com (Z.L.); qize0817@126.com (M.Z.); hbr9396@163.com (B.H.); 15924846452@163.com (W.Z.); 2 Foshan CleanEst Energy Technology Co., Ltd., Foshan 528000, China; liufeng@cleanest-energy.com (F.L.); 13610335031@163.com (W.L.); 3 Guangdong Key Laboratory for Hydrogen Energy Technologies, Foshan 528000, China; 4 Faculty of Materials Science and Chemistry, China University of Geosciences, Wuhan 430078, China

**Keywords:** corrosion resistance, tensile strength, interfacial contact resistance, hardness, hydrophobicity

## Abstract

The phase, mechanical properties, corrosion resistance, hydrophobicity, and interfacial contact resistance of Hastelloy X were investigated to evaluate its performance in proton exchange membrane fuel cells (PEMFCs). For comparison, the corresponding performance of 304 stainless steel (304SS) was also tested. Hastelloy X exhibited a single-phase face-centered cubic structure with a yield strength of 445.5 MPa and a hardness of 262.7 HV. Both Hastelloy X and 304SS exhibited poor hydrophobicity because the water contact angles were all below 80°. In a simulated PEMFC working environment (0.5 M H_2_SO_4_ + 2 ppm HF, 80 °C, H_2_), Hastelloy X exhibited better corrosion resistance than 304SS. At 140 N·cm^−2^, the interfacial contact resistance of Hastelloy X can reach as low as 7.4 mΩ·cm^2^. Considering its overall performance, Hastelloy X has better potential application than 304SS as bipolar plate material in PEMFCs.

## 1. Introduction

Hastelloy X is a typical solid-solution strengthened nickel-based high-temperature alloy with excellent oxidation resistance and high-temperature strength [1]. Hastelloy X was found to exhibit relatively good corrosion resistance in sodium borate buffer solution. [2]. And the ultimate tensile strength of Hastelloy X at 750 °C still stayed as high as 310 MPa [3]. Due to its desirable corrosion resistance and oxidation resistance, it has been widely used in high-temperature components, including gas turbine engines and gas-cooled reactors [4,5]. Another notable feature of Hastelloy X is its good fatigue resistance [6], which maintains its strength and toughness under cyclic loading conditions, making it highly durable and reducing the risk of material damage and fatigue fractures during operation [7].

Proton exchange membrane fuel cells (PEMFCs) are regarded as significant devices for addressing environmental and energy issues in the future [8]. They can directly convert chemical energy into electricity and offer advantages such as high conversion efficiency and zero emissions, making them subject to extensive research [9]. In PEMFCs, bipolar plates play crucial roles in carrying the current from the cell and preventing leakage of reactants and coolants [10,11]. They account for 45% of the stack cost and 80% of the weight [12]. Consequently, to better meet the requirements of fuel cells, an increasing number of researchers have proposed the use of metallic materials as bipolar plates.

Traditional graphite bipolar plates, despite their excellent conductivity and chemical stability, are limited in their development prospects in the field due to drawbacks such as brittleness and high cost [13]. In contrast, metal bipolar plates are considered the optimal alternative to graphite bipolar plates due to their superior mechanical durability and high conductivity [14]. Nickel-based high-temperature alloys form a dense metal oxide layer on the surface, which not only prevents oxygen from permeating into the alloy matrix but also inhibits metal dissolution, thus exhibiting outstanding oxidation resistance and corrosion resistance [15]. Furthermore, through the solid-solution strengthening mechanism, these alloys maintain high strength at elevated temperatures, demonstrating excellent high-temperature mechanical properties [16]. The 304 stainless steel (304SS) has excellent corrosion resistance as it forms oxide film on the surface [17]. When 304SS is exposed to solutions containing halide ions, especially fluoride ions, the protective film on the surface may partially break down, exposing the underlying metal, and then lead to local corrosion, including pitting and intergranular corrosion.

Currently, there is relatively limited research on the application of Hastelloy X in bipolar plates of PEMFCs. Therefore, this study aims to thoroughly investigate the electrochemical performance of Hastelloy X in a simulated PEMFC working environment and also assess its mechanical properties. For comparison, 304SS is also evaluated.

## 2. Results and Discussion

### 2.1. Phase

**Figure 1:**
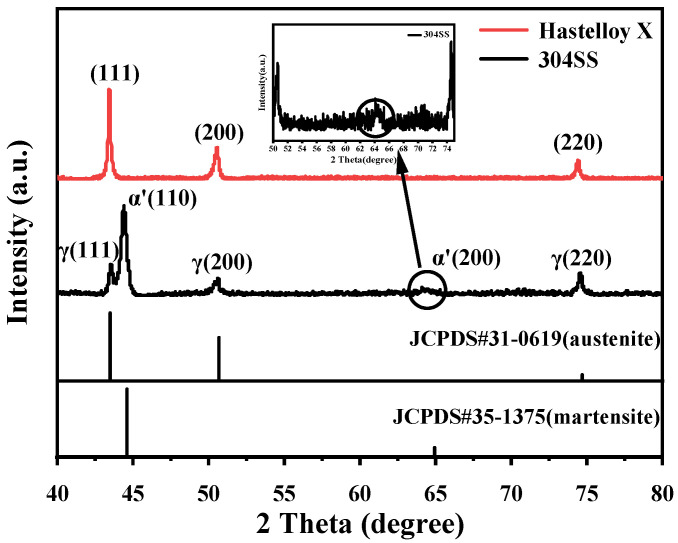
XRD patterns of Hastelloy X and 304SS.

The phase of the two alloys was examined by XRD, and the results are shown in Figure 1. The XRD pattern of Hastelloy X only has three clear characteristic peaks between 2*θ* of 40° and 80°, which demonstrates a typical face-centered cubic (FCC) polycrystalline diffraction pattern, indicating a single γ-phase [18]. According to the standard PDF card [19], 304SS exhibits austenitic and martensitic phases. This is similar to the results found by Djamel et al. [20]. At the same time, we calculated the lattice constant *a* by using the Bragg equation ($2d_{hkl}\sin\theta =n\lambda$) and the formula for the FCC crystal plane spacing $d_{hkl}=\frac{a}{\sqrt{h^{2}{+k}^{2}+l^{2}}}$. The obtained results are shown in Table 1.

### 2.2. Corrosion Resistance

**Figure 2:**
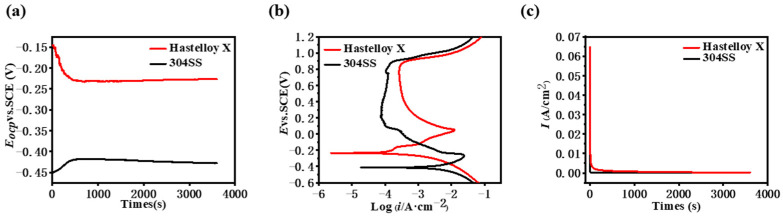
Corrosion resistance test results of Hastelloy X and 304SS (**a**) Open circuit potential curves; (**b**) potentiodynamic polarization curves; (**c**) potentiostatic polarization curves.

Figure 2a shows the curve of the open circuit potential (*E*_ocp_) of Hastelloy X in a 0.5 M H_2_SO_4_ +2 ppm HF at 80 °C. The shift of the open circuit potential towards a negative potential is probably due to the dissolution of the passivation layer. The *E*_ocp_ of Hastelloy X gradually decreases over time and then stabilizes at approximately −0.23 V after 500 s of immersion. In contrast, the potential of 304SS shifts towards a positive potential over time. In order to assess the corrosion resistance of Hastelloy X in a simulated PMEFC working environment, a potentiodynamic potential polarization method was used. Figure 2b shows the electrochemical polarization curves, and the corresponding corrosion parameters are also summarized in Table 2. It can be seen from Figure 2b that Hastelloy X undergoes a transition from cathodic polarization, activation, activation to passivation, and finally the formation of a passivation film, and then the passivation film dissolves. In the cathodic polarization region (−0.6, −0.23) V, the current density decreases with increasing potential. When the scanning potential exceeds −0.23 V, the current density increases with increasing potential and then decreases with increasing potential after exceeding +0.05 V, finally stabilizing at 2.84 × 10^−4^ A·cm^−2^. As the anodic polarization potential continues to increase, the anodic current density increases rapidly, indicating that in the range of (+0.84, +1.20) V, the passivation film begins to break and loses its passivation capability completely. In contrast, 304SS exhibits a much higher corrosion current, demonstrating its poor corrosion resistance in a simulated PEMFC working environment. Figure 2c shows potentiostatic polarization curves in a simulated PEMFC working environment for further assessing the corrosion resistance and stability of Hastelloy X and 304SS. As can be seen from the graph, the current density drops rapidly in the initial phase after voltage application and stabilizes after 30 s, indicating that Hastelloy X has good stability in a simulated PEMFC working environment. In order to further improve its electrochemical properties, proper surface modification techniques can be used to improve corrosion resistance [11].

**Figure 3:**
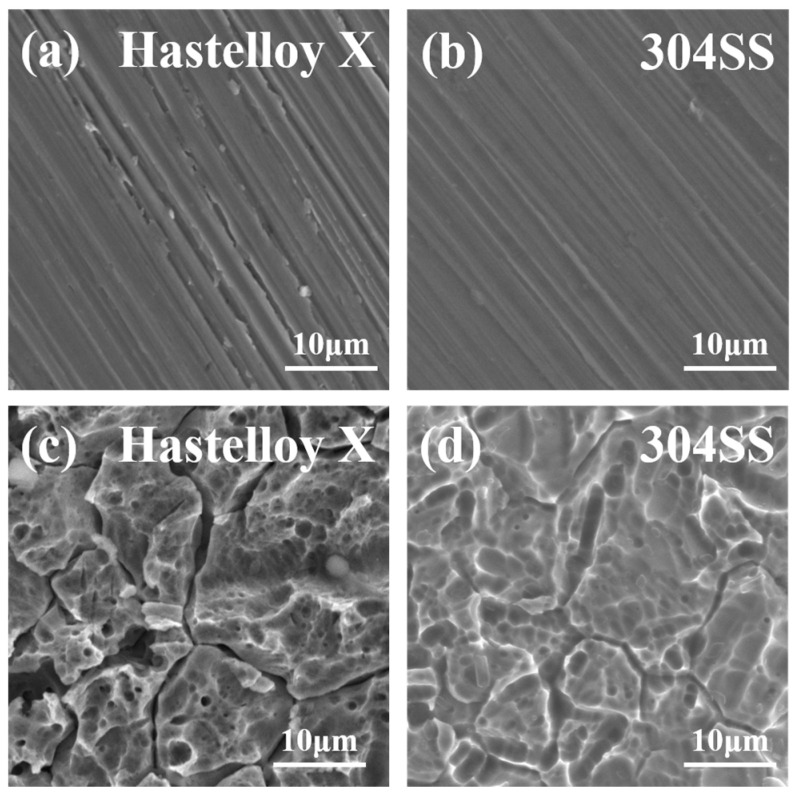
SEM images of the surface of Hastelloy X and 304SS (**a**,**b**) before and (**c**,**d**) after electrochemical testing.

The surface morphology of Hastelloy X and 304SS before and after electrochemical testing was observed using SEM, and the images are shown in Figure 3. Before electrochemical testing, the surfaces of both alloys showed machined filamentary morphology (Figure 3a,b). After the testing, pitting appeared on the surfaces of Hastelloy X and 304SS (Figure 3c,d). As shown in Table 3 and Table 4, the atomic ratio of Cr to Mo decreased in both samples, and oxygen atoms were present, probably due to the formation of Cr and Mo oxides during the corrosion process. For 304SS, the presence of Cr oxide in its surface passivation film is attributed to the selective dissolution of Fe during the passivation process, resulting in the enrichment of Cr within the passivation film [21].

**Figure 4:**
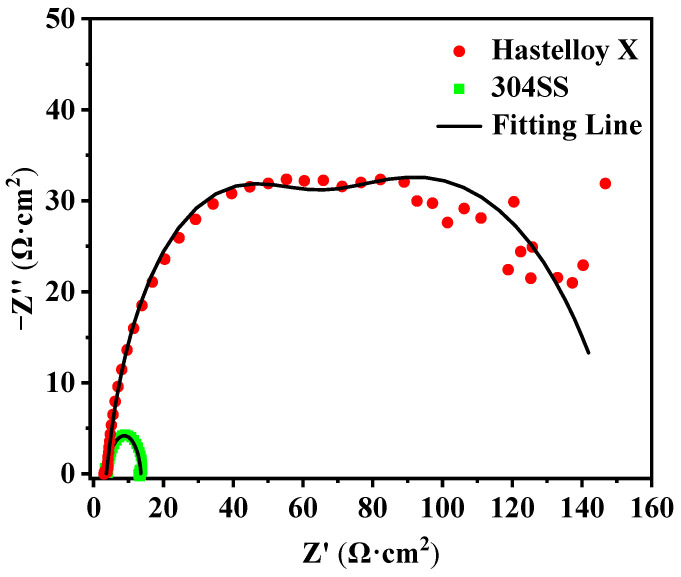
Nyquist plot of the alloys after corrosion.

**Figure 5:**
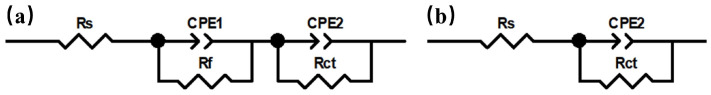
Equivalent circuits for fitting the impedance diagram of (**a**) Hastelloy X and (**b**) 304SS.

Electrochemical impedance spectroscopy (EIS) is another powerful technique to evaluate corrosion protection performance. Compared to 304SS, Hastelloy X showed a positive shift in impedance arc radius in both the high and medium frequency ranges (Figure 4), indicating that Hastelloy X is more resistant to corrosion. The equivalent circuit model as shown in Figure 5 was proposed to fit the EIS data based on the above analysis. Rs, CPE1, Rf, CPE2, and Rct are the solution resistance, oxide film protection capacitance, oxide film protection resistance, double layer capacitance, and charge transfer resistance, respectively. Notably, since the capacitor loop in Nyquist plots were not perfect semicircles, a constant phase element is employed to replace the ideal capacitor.

The fitting impedance parameters are summarized in Table 5. The results show that Hastelloy X has higher Rct value, which demonstrates better corrosion resistance than that of 304SS. Furthermore, it is well known that CPE is an important indicator of the corrosion area of metals, and the smaller the CPE value, the smaller the area of corrosion indicated. From Table 5, an elevated order of CPE2 can be implied: Hastelloy X (1.4 × 10^−3^ Ω^−1^·cm^−2^·s^n^) < 304SS (1.1 × 10^−2^ Ω^−1^·cm^−2^·s^n^), suggesting the smallest corrosion area for Hastelloy X.

### 2.3. Mechanical Properties

**Figure 6:**
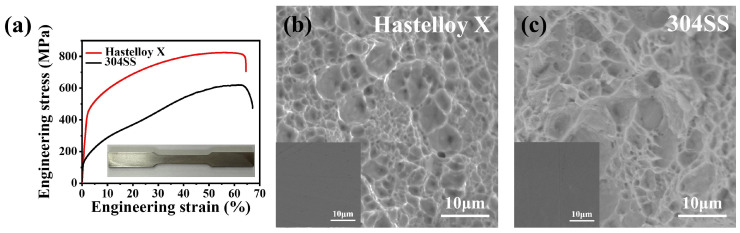
Mechanical testing results of Hastelloy X and 304SS. (**a**) Tensile stress–strain curves of alloys; SEM images of the fracture surface of (**b**) Hastelloy X and (**c**) 304SS (left bottom inset in panels (**b**,**c**) is the SEM image of the corresponding alloy before tensile testing).

The tensile stress–strain curves of Hastelloy X and 304SS are shown in Figure 6a, and the corresponding mechanical properties are summarized in Table 6. The tensile data of Hastelloy X are similar to the results obtained by Ghiaasiaan et al. [22]. The yield strength of Hastelloy X is 445 MPa, which ishigher than that of 304SS (127 MPa), indicating that Hastelloy X has a higher resistance to stress. The ultimate tensile strength of Hastelloy X is 823.9 MPa, surpassing the 616.3 MPa of 304SS, demonstrating its ability to withstand higher tensile forces. The uniform elongation is an indicator of the plastic deformation capacity of a material. The uniform elongation for Hastelloy X is 56.5%, slightly lower than the 61.4% of 304SS. Higher uniform elongation usually indicates better ductility. The elastic modulus, which represents the material’s response to stress, is 203.8 GPa for Hastelloy X, which is lower than that of 304SS (273.7 GPa), indicating that Hastelloy X is more flexible. Hardness is an indicator of a material’s resistance to localized plastic deformation and surface damage. The hardness of Hastelloy X is 262.7 HV, which is slightly higher than that of 304SS (215.3 HV). Higher hardness usually suggests better wear resistance [23]. The higher hardness of Hastelloy X may be due to it being a solid-solution strengthened high-temperature alloy without reinforcing precipitates like the gamma phase. Consequently, Hastelloy X obviously outperforms 304SS in yield strength, ultimate tensile strength, and hardness, indicating that Hastelloy X has excellent mechanical properties. Due to the use of 304SS produced by different manufacturers, the mechanical properties of 304SS differ slightly from those of previous reports [24].

The fracture morphologies of Hastelloy X and 304SS after tensile testing were observed using SEM to analyze their fracture mechanism. Both of the fracture surfaces of Hastelloy X and 304SS exhibit numerous dimples and continuous fiber networks, as shown in Figure 6b,c, indicating typical ductile fracture and implying good ductility. This observation is consistent with the uniform elongation values of 56.5% and 61.4% for Hastelloy X and 304SS, respectively. Particularly, the dimple size of 304SS is larger than that of Hastelloy X, indicating that 304SS undergoes greater plastic deformation before fracture. This characteristic aligns with the higher elongation capability of 304SS.

### 2.4. Hydrophobicity

**Figure 7:**
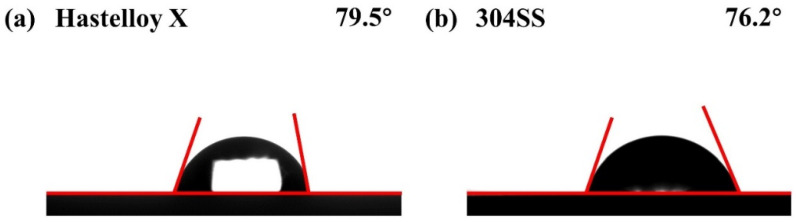
Water contact angle of (**a**) Hastelloy X and (**b**) 304SS.

For bipolar plates, water management is crucial to improving the performance of PEMFCs. It helps to prevent flooding in the channels and gas diffusion layer, thereby avoiding deterioration of mass transport and the occurrence of corrosion. Therefore, hydrophobic bipolar plates with high water contact angles are preferable to PEMFCs. Water contact angle (WCA) testing was employed to evaluate the hydrophobicity of the two alloys, and the measurement results are shown in Figure 7. Hastelloy X exhibits a WCA value of 79.5°, which is larger than that of 304SS (76.2°). Meanwhile, the hydrophobicity of Hastelloy X was significantly better than in our previous studies on Hastelloy C-276 and Hastelloy B [25]. However, both of the WCA values of Hastelloy X and 304SS are less than 80°, indicating poor hydrophobicity.

### 2.5. Interfacial Contact Resistance (ICR)

**Figure 8:**
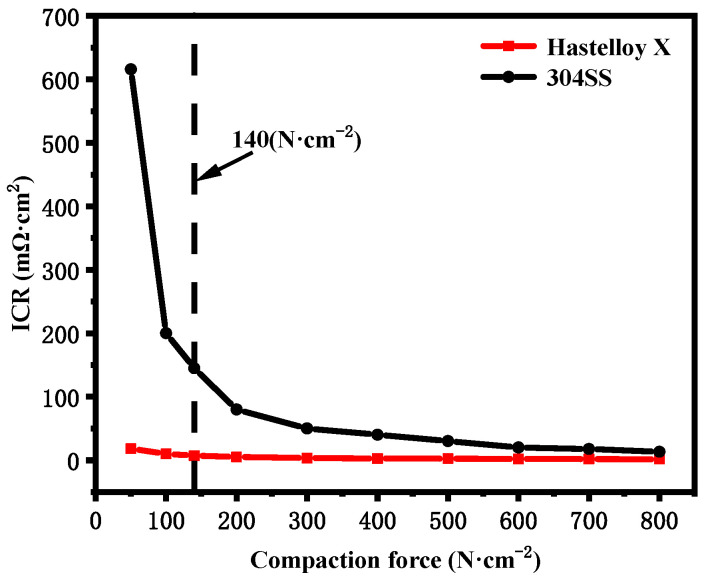
ICR of the alloys at different levels of compaction force.

ICR is a critical parameter that significantly impacts the performance of bipolar plates. Maintaining a low ICR is crucial for the materials utilized in these plates, as a high ICR can lead to a reduced lifespan of PEMFCs. Figure 8 illustrates the correlation between compaction force and ICR values for Hastelloy X and 304SS. Compared to Hastelloy X, 304SS demonstrates higher ICR values. The ICR values of 304SS [26] decrease with increasing compaction force and stabilize at higher compaction forces. This phenomenon can be attributed to the fact that, under high load conditions, the actual contact area between the interfaces expands, resulting in improved conductivity. Table 7 provides the ICR values of the two alloys at a compaction force of 140 N·cm^−2^ [27]. The ICR of 304SS is higher than that of Hastelloy X, with a value of 144.8 mΩ·cm^2^. This elevated ICR in 304SS can be attributed to the presence of surface oxide layers composed of iron, nickel, and chromium oxides. On the other hand, Hastelloy X demonstrates excellent conductivity, with an ICR of 7.4 mΩ·cm^2^ at 140 N·cm^−2^, which satisfies the 2025 Department of Energy (DOE) target of less than 10 mΩ·cm^2^ [28]. The very low ICR of Hastelloy X can be attributed to the addition of Cr^3+^ to oxides of Ni or Co [29], which largely increases the conductivity of the passivation film on the surface of Hastelloy X.

## 3. Materials and Methods

**Figure 9:**
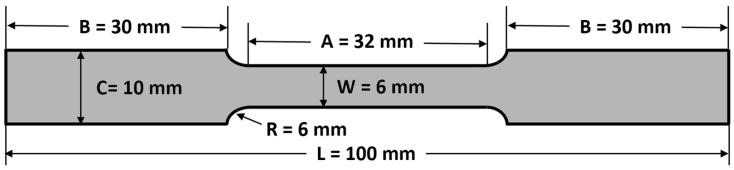
Geometry of tensile samples.

Commercially available Hastelloy X was used. The samples were prepared according to the standard test method for tensile testing of metallic materials [30]. The samples were respectively cut into dog bone shapes (Figure 9), square shapes with dimensions of 20 mm × 20 mm × 2 mm and 50 mm × 50 mm × 2 mm, using wire cutting. The samples used for electrochemical performance testing were circular discs with a diameter of 15 mm and a thickness of 2 mm. All samples were polished using SiC paper before testing.

The surface morphology of the samples before and after corrosion was observed using a Hitachi SU-1500 scanning electron microscope (SEM, Tokyo, Japan), and composition analysis was conducted using an energy-dispersive X-ray spectrometer (Horiba, Tokyo, Japan, EMAX x-act model). The scanning area of each SEM image was 63.84 μm × 47.88 μm. To investigate the phase composition of Hastelloy X, we utilized a TD-3500 X-ray diffractometer (XRD) produced by Tongda Corporation (Dandong, China). The X-ray source employed the characteristic Kα line (λ = 0.15406 nm) of a Cu target. The scanning angle range was set from 40° to 80° with a step size of 0.02° and a scanning speed of 1°/min.

Electrochemical testing was conducted using the CorrTest CS150M electrochemical workstation (Wuhan, China). Platinum foil was used as the counter electrode, a saturated calomel electrode (SCE) served as the reference electrode, and the sample acted as the working electrode. Each sample was immersed into a simulated PEMFC working environment (0.5 M H_2_SO_4_ + 2 ppm HF, 80 °C, 20 mL/min H_2_) for one hour to reduce experimental errors. Open circuit potential (OCP) testing was then performed until the entire system stabilized. The potentiodynamic potential polarization was scanned from −0.6 V to +1.2 V at a scan rate of 0.2 mV/s to evaluate the corrosion resistance of the alloys with a 60 min potentiostatic polarization test. The corrosion potential and corrosion current density were obtained using the Tafel extrapolation method. The frequency range for EIS testing was set at 10^5^~10^−2^ Hz, and a perturbation voltage of 20 mV was utilized. To ensure reliability, all tests were replicated three times.

Tensile testing was conducted using an MTS Landmark testing machine at a rate of 1 mm/min. A video extensometer with a gauge length of 25 mm was used to record displacement. The samples were tested three times to evaluate the variation in tensile performance, and the fracture surface was observed using SEM. Hardness testing was performed using an HXS-1000tac microhardness tester (Zhongkekaihua Technology Development Co., Ltd., Lanzhou, China) with a load of 200 N and a dwell time of 15 s, following the test method [31] for microindentation hardness of materials. To reduce measurement errors, the hardness tester was calibrated with a standard hardness block (238 HV_0.2_), and 10 measurements were taken from each sample.

The hydrophobicity of the samples was tested using a JC2000D1B water contact angle measurement instrument (YIMA, Hong Kong, China). To minimize errors, the contact angle value was determined as the average of 10 measurements.

The ICR was determined following the method proposed by Jin et al. [32]. Carbon paper was placed on both sides of the sample and placed between two copper plates with gold coating. The Testometric M350-CT universal testing machine (The Testometric Co. Ltd., Rochdale, UK) provided compressive force and recorded data, while the resistance between the copper electrodes was measured using the ZY9858 digital micro-ohmmeter(Shanghai Instrumentation Co., Shanghai, China). The sample resistance was tested three times to reduce errors.

## 4. Conclusions

We conducted a comprehensive study on the performances of Hastelloy X and 304SS as potential bipolar plate materials of PEMFCs. Hastelloy X exhibited excellent mechanical performance due to its single-phase FCC structure. However, significant differences were observed in their corrosion resistance and ICR. In a simulated PEMFC working environment, Hastelloy X demonstrated better corrosion resistance compared to 304SS, although it still fell short of meeting the 2025 DOE target. Fortunately, under a compaction force of 140 N·cm^−2^, Hastelloy X achieved an ICR as low as 7.4 mΩ·cm^2^, which can meet the 2025 DOE target. Therefore, Hastelloy X shows excellent mechanical properties and conductivity, making it a potential candidate material for PEMFC metal bipolar plates.

## Figures and Tables

**Table 1:** X-ray diffraction angles of peaks and lattice constant of the alloys.

Alloys	*θ*_111_ (°)	*θ*_200_ (°)	*θ*_220_ (°)	*a* (nm)
Hastelloy X	21.77	25.35	37.27	0.3603
304SS	21.74	25.34	37.34	0.3464

**Table 2:** Electrochemical parameters of the alloys.

Alloys	*E*_ocp_ (V)	*E*_corr_ (V)	*I*_corr_ (A·cm^−2^)
Hastelloy X	−0.23	−0.23	(1.01 ± 0.01) × 10^−4^
304SS	−0.45	−0.34	(7.36 ± 0.03) × 10^−3^

**Table 3:** Chemical composition of Hastelloy X (at. %).

	Ni	Fe	Cr	Mo	Co	Al	O
Before electrochemical testing	49.28	20.10	23.36	5.09	1.96	0.2	/
After electrochemical testing	52.04	16.84	15.79	3.92	1.97	0.03	9.40

**Table 4:** Chemical composition of 304SS (at. %).

	Ni	Fe	Cr	Si	O
Before electrochemical testing	7.23	72.62	18.96	0.64	/
After electrochemical testing	5.95	61.41	16.47	0.62	15.55

**Table 5:** EIS analyzed results of the alloys.

Alloys	CPE1/Ω^−1^·cm^−2^·s^n^	n1	Rf/Ω·cm^2^	CPE2/Ω^−1^·cm^−2^·s^n^	n2	Rct/Ω·cm^2^
Hastelloy X	(1.9 ± 0.1) × 10^−3^	0.651 ± 0.002	100.7 ± 5.2	(1.4 ± 0.1) × 10^−3^	0.920 ± 0.012	47.58 ± 0.43
304SS	/	/	/	(1.1 ± 0.2) × 10^−2^	0.915 ± 0.023	9.57 ± 0.50

**Table 6:** Mechanical properties of Hastelloy X and 304SS.

Alloys	Yield Strength 0.2% Offset (MPa)	Ultimate Tensile Strength (MPa)	Uniform Elongation (%)	Elastic Modulus (GPa)	Hardness (HV)
Hastelloy X	445.5 ± 2.3	823.9 ± 5.1	56.5 ± 1.3	203.8 ± 5.7	262.7 ± 1.2
304SS	127.3 ± 4.6	616.3 ± 4.3	61.4 ± 2.5	273.7 ± 3.8	215.3 ± 6.5

**Table 7:** ICR of the alloys at 140 N·cm^−2^.

Alloys	ICR (mΩ·cm^2^)
Hastelloy X	7.4 ± 0.3
304SS	144.8 ± 5.4

## Data Availability

Data are contained within the article.

## References

[1] Zhang W., Liu F., Liu F., Huang C., Zheng H., Zhang Q., Zheng Y., Gao J. (2022). Microstructural evolution and cracking behavior of Hastelloy X superalloy fabricated by laser directed energy deposition. J. Alloys Compd..

[2] Kong D., Ni X., Dong C., Zhang L., Yao J., Man C., Wang L., Xiao K., Li X. (2019). Anisotropic response in mechanical and corrosion properties of hastelloy X fabricated by selective laser melting. Constr. Build. Mater..

[3] Karapuzha A.S., Fraser D., Schliephake D., Dietrich S., Zhu Y., Wu X., Huang A. (2023). Room and elevated temperature tensile and fatigue behaviour of additively manufactured Hastelloy X. Mater. Sci. Eng. A.

[4] Guo B., Zhang Y., Yang Z., Cui D., He F., Li J., Wang Z., Lin X., Wang J. (2022). Cracking mechanism of Hastelloy X superalloy during directed energy deposition additive manufacturing. Addit. Manuf..

[5] Yin Y., Li H., Pan S., Zhang J., Han Q., Yang S. (2022). Electrochemical behaviour of passivation film formed on SLM-fabricated Hastelloy X superalloy surface in 10 wt% NaNO_3_ solution. Corros. Sci..

[6] Yoon D., Heo I., Kim J., Chang S., Chang S. (2019). Hold Time-low cycle fatigue behavior of nickel based Hastelloy X at elevated temperatures. Int. J. Precis. Eng. Manuf..

[7] Kim I., Choi B., Jung J., Do J., Jo C. (2015). Effect of microstructural characteristics on the low cycle fatigue behaviors of cast Ni-base superalloys. Mater. Charact..

[8] Song Y., Zhang C., Ling C.-Y., Han M., Yong R.-Y., Sun D., Chen J. (2020). Review on current research of materials, fabrication and application for bipolar plate in proton exchange membrane fuel cell. Int. J. Hydrogen Energy.

[9] Huang P., Chen Z., Zhang J., Wu M., Liu Y., Zhang F., Chen Y., Chen X. (2022). Stainless steel bipolar plate fuel cell with different flow field structures prepared by laser additive manufacturing. Int. J. Heat Mass Transf..

[10] Li H., Guo P., Zhang D., Liu L., Wang Z., Ma G., Xin Y., Ke P., Saito H., Wang A. (2020). Interface-induced degradation of amorphous carbon films/stainless steel bipolar plates in proton exchange membrane fuel cells. J. Power Sources.

[11] Ma G., Zhang D., Guo P., Li H., Xin Y., Wang Z., Wang A. (2022). Phase orientation improved the corrosion resistance and conductivity of Cr2AlC coatings for metal bipolar plates. J. Mater. Sci. Technol..

[12] Yu Y., Li H., Wang H., Yuan X.-Z., Wang G., Pan M. (2012). A review on performance degradation of proton exchange membrane fuel cells during startup and shutdown processes: Causes, consequences, and mitigation strategies. J. Power Sources.

[13] Asri N.F., Husaini T., Sulong A.B., Majlan E.H., Daud W.R.W. (2017). Coating of stainless steel and titanium bipolar plates for anticorrosion in PEMFC: A review. Int. J. Hydrogen Energy.

[14] Gao P., Xie Z., Wu X., Ouyang C., Lei T., Yang P., Liu C., Wang J., Ouyang T., Huang Q. (2018). Development of Ti bipolar plates with carbon/PTFE/TiN composites coating for PEMFCs. Int. J. Hydrogen Energy.

[15] Wang X.-Z., Jiang Y., Wang Y., Ye C., Du C.-F. (2022). Probing the tribocorrosion behaviors of three nickel-based superalloys in sodium chloride solution. Tribol. Int..

[16] Shahwaz, Nath P., Sen I. (2022). A critical review on the microstructure and mechanical properties correlation of additively manufactured nickel-based superalloys. J. Alloys Compd..

[17] Zhang H., Wu Z., Chen Y., Feng K., Yan H., Song H., Luo C., Hu Z. (2024). Real-time monitoring of the corrosion behaviour of the 304SS in HCl solution using BPNN with joint image recognition and electrochemical noise. Corros. Sci..

[18] Jinoop A., Paul C., Bindra K. (2019). Laser assisted direct energy deposition of Hastelloy-X. Opt. Laser Technol..

[19] Saeidi K., Gao X., Zhong Y., Shen Z.J. (2015). Hardened austenite steel with columnar sub-grain structure formed by laser melting. Mater. Sci. Eng. A.

[20] Kaoumi D., Liu J. (2018). Deformation induced martensitic transformation in 304 austenitic stainless steel: In-situ vs. ex-situ transmission electron microscopy characterization. Mater. Sci. Eng. A.

[21] Wang J., Li W., Yang H., Huang H., Ji S., Ruan J., Liu Z. (2020). Corrosion behavior of CoCrNi medium-entropy alloy compared with 304 stainless steel in H2SO4 and NaOH solutions. Corros. Sci..

[22] Ghiaasiaan R., Muhammad M., Gradl P.R., Shao S., Shamsaei N. (2021). Superior tensile properties of Hastelloy X enabled by additive manufacturing. Mater. Res. Lett..

[23] Ogihara H., Wang H., Saji T. (2014). Electrodeposition of Ni–B/SiC composite films with high hardness and wear resistance. Appl. Surf. Sci..

[24] Liu Z.M., Zhong J.C., Zhang M., Hou B.R., Zhang W.M., Zhao C.W. (2023). Mechanical properties of Hastelloy X and C-276 and their application in hydrogen fuel cell bipolar plates. Mech. Eng..

[25] Zhong J., Hou B., Zhang W., Guo Z., Zhao C. (2023). Investigation on the physical and electrochemical properties of typical Ni-based alloys used for the bipolar plates of proton exchange membrane fuel cells. Heliyon.

[26] Xuan J., Xin Y., Xu L., Guo M., Huang L., Zhang Y., Zhao Y., Liu Y., Li L., Xue L. (2023). Effects of fluoride ions on corrosion performance and surface properties of SS304 in simulated PEMFC cathodic environments. Renew. Energy.

[27] Fuel Cell Technical Team Roadmap (2017). https://www.energy.gov/eere/vehicles/us-drive-partnership-plan-roadmaps-and-accomplishments.

[28] Yin Q., Zhang K., Fu X.-Z., Wang X.-Z., Luo J.-L. (2022). Rapid coating preparation strategy for chromium nitride coated titanium bipolar plates of proton exchange membrane fuel cells. Int. J. Hydrogen Energy.

[29] Liu T., Diao P. (2020). Nickel foam supported Cr-doped NiCo_2_O_4_/FeOOH nanoneedle arrays as a high-performance bifunctional electrocatalyst for overall water splitting. Nano Res..

[30] Kannan A.R., Shanmugam N.S., Palguna Y., Girinath B., Lee W., Yoon J. (2023). Effect of double-side welding on the microstructural characteristics and mechanical performance of dissimilar AA6061-AA5052 aluminium alloys. Mater. Lett..

[31] Li D.W., Liu J.X., Sun Y.T., Huang W.Q., Li N., Yang L.H. (2023). Microstructure and mechanical degradation of K403 Ni-based superalloy from ultra-long-term serviced turbine blade. J. Alloys Compd..

[32] Jin J., Liu H., Zheng D., Zhu Z. (2018). Effects of Mo content on the interfacial contact resistance and corrosion properties of CrN coatings on SS316L as bipolar plates in simulated PEMFCs environment. Int. J. Hydrogen Energy.

